# Irrational Delay Revisited: Examining Five Procrastination Scales in a Global Sample

**DOI:** 10.3389/fpsyg.2017.01927

**Published:** 2017-11-03

**Authors:** Frode Svartdal, Piers Steel

**Affiliations:** ^1^Department of Psychology, UiT The Arctic University of Norway, Tromsø, Norway; ^2^Organizational Behaviour and Human Resources, University of Calgary, Calgary, AB, Canada

**Keywords:** procrastination, procrastination scale, measurement, confirmatory factor analysis, item response theory

## Abstract

Scales attempting to measure procrastination focus on different facets of the phenomenon, yet they share a common understanding of procrastination as an unnecessary, unwanted, and disadvantageous delay. The present paper examines in a global sample (*N* = 4,169) five different procrastination scales – Decisional Procrastination Scale (DPS), Irrational Procrastination Scale (IPS), Pure Procrastination Scale (PPS), Adult Inventory of Procrastination Scale (AIP), and General Procrastination Scale (GPS), focusing on factor structures and item functioning using Confirmatory Factor Analysis and Item Response Theory. The results indicated that The PPS (12 items selected from DPS, AIP, and GPS) measures different facets of procrastination even better than the three scales it is based on. An even shorter version of the PPS (5 items focusing on irrational delay), corresponds well to the nine-item IPS. Both scales demonstrate good psychometric properties and appear to be superior measures of core procrastination attributes than alternative procrastination scales.

## Introduction

Measurement of self-reported procrastination in tests and questionnaires focuses on different areas in which unnecessary delay expresses itself. As per Goal Phase Theory (Steel and Weinhardt, 2017), aside from goal attainment itself, motivation can be broken down into a decisional stage, a planning stage and a goal striving or implementation stage, with people capable of procrastinating in each or all of them. Although these aspects of procrastination are closely related, they may still be differentiated and are often measured by different instruments. Thus, the Decisional Procrastination Scale (DPS, five items related to procrastination; Mann, 1982, unpublished; Mann et al., 1997) focuses on delay in planning and decision making, whereas general procrastination scales such as the General Procrastination Scale (GPS; Lay, 1986) address implemental or behavioral delay. McCown and Johnson’s Adult Inventory of Procrastination Scale (AIP; McCown et al., 1989) completes this picture by including summary items related to promptness, meeting deadlines, and timeliness.

To identify the core attributes of procrastination, Steel (2010) suggested two new instruments. First is the Irrational Procrastination Scale (IPS), which consists of nine items focusing on implemental attributes of procrastination with an emphasis on “irrational” delay, “irrational” referring to voluntary delay despite expecting it to be disadvantageous. Second is the Pure Procrastination Scale (PPS, 12 items), which is based on items from existing and somewhat diverse procrastination scales (i.e., the DPS, GPS, and AIP) selected after factor analyses of responses from more than 4000 respondents. Exploratory and confirmatory factor analyses indicated a three-factor solution for the items contained in the instruments, with the first factor addressing habitual or problematic delay. This factor contained 14 items of which 12 of the highest loading were selected for the PPS. All three established scales were represented in this selection. In effect, the PPS is a mix of established scales measuring somewhat different aspects of procrastination, but still loading high on a factor that addresses implemental delay. Unsurprisingly, therefore, the PPS and IPS correlate highly, *r* = 0.87 (Steel, 2010).

Despite the similarity between the IPS and the PPS, examination of the items selected for the PPS indicates a broader understanding of “delay” compared to the IPS. **Table 1** shows the items of both scales. Whereas the IPS items predominantly focus on implemental delay, the PPS also includes items related to decisional delay and timeliness. Implicitly, the PPS therefore assumes that decisional and behavioral delay, as well as delay in promptness and timeliness, are closely related. This was not substantiated in the original article (Steel, 2010), neither was the hypothesis that the PPS in fact measures a unidimensional construct related to problematic and habitual delay.

**Table 1:** Irrational Procrastination Scale (IPS) and Pure Procrastination Scale (PPS) Items.

Scale	Item	Origin
IPS	(1) I put things off so long that my well-being or efficiency unnecessarily suffers	
IPS	(2) If there is something I should do, I get to it before attending to lesser tasks (R)	
IPS	(3) My life would be better if I did some activities or tasks earlier	
IPS	(4) When I should be doing one thing, I will do another	
IPS	(5) At the end of the day, I know I could have spent the time better	
IPS	(6) I spend my time wisely (R)	
IPS	(7) I delay tasks beyond what is reasonable	
IPS	(8) I procrastinate	
IPS	(9) I do everything when I believe it needs to be done (R)	
PPS	(1) I delay making decision until it’s too late.	DPQ4
PPS	(2) Even after I make a decision I delay acting upon it.	DPQ2
PPS	(3) I waste a lot of time on trivial matters before getting to the final decisions.	DPQ1
PPS	(4) In preparation for some deadlines, I often waste time by doing other things.	GPS12
PPS	(5) Even jobs that require little else except sitting down and doing them.	GPS7
	I find that they seldom get done for days.	
PPS	(6) I often find myself performing tasks that I had intended to do days before	GPS1
PPS	(7) I am continually saying “I’ll do it tomorrow.”	GPS19
PPS	(8) I generally delay before starting on work I have to do.	GPS9
PPS	(9) I find myself running out of time.	AIP10
PPS	(10) I don’t get things done on time.	AIP5
PPS	(11) I am not very good at meeting deadlines.	AIP9
PPS	(12) Putting things off till the last minute has cost me money in the past.	AIP15

*(R) denoted items are reversed scored. DPS, Decisional Procrastination Scale; GPS, General Procrastination Scale; AIP, Adult Inventory of Procrastination Scale*.

Subsequent examinations of the PPS have obtained somewhat diverging results regarding factor structure of this scale. For example, an assessment of a translated PPS for French-speaking individuals (Rebetez et al., 2014) indicated that the PPS should be reduced to 11 instead of 12 items, the remaining items comprising a two-factor solution with items 1–8 and items 9–11 loading on different constructs, “voluntary delay” and “observed delay.” A Swedish study (Rozental et al., 2014) obtained a different two-factor solution for the PPS, one factor being related to delaying decision making, not meeting deadlines, and missing appointments (items 1–3 and 9–12), whereas the other was associated with starting late, lagging behind, and wasting time (items 4–8). Neither of these suggestions addressed the fact that the PPS consists of items from three established procrastination scales, each set of items tapping into somewhat different aspects of problematic delay (e.g., decisional and implemental). Hence, Svartdal et al. (2016), in a European study with 2893 student and employee participants from six countries, examined the hypothesis that the PPS might measure multiple aspects. Confirmatory factor analyses indicated poor fits for the two-factor solutions discussed, as well as for a one-factor solution implied by Steel (2010), but a good fit for a three-factor solution addressing decisional delay (PPS items 1–3), implemental delay (items 4–8), and lateness/timeliness (items 9–12). The middle part of PPS (items 4–8) demonstrated considerable cross-national and subgroup stability whereas the latter part (items 9–12) seemed to vary both between nations and students vs. employees. This may indicate that the middle part of the PPS addresses core properties of problematic procrastination whereas the latter part is more closely related to procrastination in a cultural and situational context.

As for the IPS, this scale attempts to measure a single construct, “irrational delay” (Steel, 2010). Research has subsequently confirmed this (e.g., Svartdal et al., 2016), although the three reversed items of the scale (items 2, 6, and 9) seem to measure the construct somewhat less optimally compared to the others and have even been suggested to load on a different factor (Rozental et al., 2014).

The remaining procrastination scales discussed in this paper, DPS, AIP, and GPS, have all been widely used, but surprisingly few studies have assessed their psychometric properties. For example, Lay (1986) proposed the GPS as a scale measuring a unidimensional construct procrastination, but few studies have examined this scale psychometrically using confirmatory factor analysis (CFA). One study (Argiropoulou and Ferrari, 2015) using a Greek sample suggested, in contrast to the original ambition about unidimensionality, a two-factor solution (i.e., *delay* and *procrastination domains*). A German study, testing the student version of the GPS, could not confirm an one-factorial structure and instead proposed a reduced version – GPS-K – consisting of items 1, 2, 7, 12, 14, 15, 18, 19, and 20 (Klingsieck and Fries, 2012). These items (except items 2 and 14) are identical in the general version of GPS. As for the AIP, this scale originally hypothesized a single latent construct, procrastination. Very few studies have examined the AIP using CFA, an exception being Mariani and Ferrari (2012), reporting support for a single-factor latent model in an Italian sample. Finally, the DPS (a subset of 31/22 items in the Flinders/Melbourne decision-making questionnaire; Mann et al., 1997) measures decisional procrastination. Mann et al. (1997) found that the procrastination subscale demonstrated good fit within the revised Melbourne decision-making model. Little is known regarding the factor structure of this subscale *per se*, but Mariani and Ferrari (2012) reported support for a unidimensional factor solution in an Italian sample.

When comparing the various scales, it should be remembered that whereas the DPS intended to measure decisional delay, the AIP and GPS are general procrastination scales measuring a unidimensional latent construct, procrastination, in the much same way as intended by the PPS and IPS. However, as is seen in **Table 2**, the various scales contain both decisional or implemental procrastination items, as well as items related to lateness/timeliness, somewhat sporadically. Evaluating the scale items at face value, the GPS and IPS both have their focus on implemental delay, whereas most AIP items address timeliness and lateness. The PPS, being composed of items from DPS, GPS, and AIP, thus appears to be a hybrid scale with a broad focus not matched by any of the other scales. Also note that both the AIP (20 items) and GPS (15 items) are relatively comprehensive instruments. Because procrastination scales are often administered with scales measuring other constructs, shorter instruments with comparable or even better psychometric qualities compared to the full scales contribute to overall reduction of survey length and should be used if possible (Stanton et al., 2002; Rogelberg and Stanton, 2007).

**Table 2:** The procrastination scales and their different foci Of decisional, implemental, or timeliness/lateness.

Scale (items)	Decisional	Implemental	Timeliness, lateness
DPS (5)	1, 3, 4, 5	2	
AIP (15)	11, 3	7, 8, 13	1, 2, 4, 5, 6, 9, 10, 12, 14, 15
GPS (20)	8	Most	2
IPS (9)		1, 2, 3, 4, 5, 6, 7, 8, 9	
PPS (12)	1, 2, 4 (from DPS)	1, 7, 9, 12, 19 (from GPS)	5, 9, 10, 15 (from AIP)

*DPS, Decisional Procrastination Scale; AIP, Adult Inventory of Procrastination Scale; GPS, General Procrastination Scale; IPS, Irrational Procrastination Scale (IPS); PPS, Pure Procrastination Scale*.

### The Present Study

The present paper examines the PPS and IPS, as well as the complete DPS, AIP, and GPS instruments in a global data set with 4,169 participants. Using Confirmatory Factor Analyses (CFI) and Item Response Theory (IRT), we compare the different scales, examine their factor structures, and suggest simplified scales to measure procrastination. We also assess measurement invariance over gender and age, internal reliability, as well as correlations between the instruments.

The first purpose of the present study was to examine the possibility that the PPS addresses three rather different facets of procrastination (Svartdal et al., 2016), and that the middle five items of the PPS correspond to IPS in measuring “irrational” delay. As the GPS seems to measure this construct also, the full GPS as well as reduced versions were examined. Lay (1986) hypothesized the GPS to measure a unidimensional construct, but as mentioned, subsequent studies have not supported this assumption and have instead suggested different factor structures (Argiropoulou and Ferrari, 2015) or a reduced version (Klingsieck and Fries, 2012). We examine these possibilities, the latter being particularly interesting because the five implemental items of the PPS (4–8) are in fact GPS items. Thus, the possibility that the GPS could be reduced to five items is tested. Finally, the DPS and AIP were also examined. Recall from **Table 2** that the DPS focuses on decisional procrastination, whereas the AIP contains several items that focus on timeliness and lateness. As the PPS includes items from both these scales, we ask how these PPS items perform compared to the full scales. In effect, we pursue the possibility that the DPS and AIP could be reduced to fewer items, likely corresponding the PPS items, and in consequence that the PPS could replace the DPS, AIP, and the GPS scales.

## Materials and Methods

### Sample

The sample consisted of 4169 respondents (57.4% women) completing an online survey. Mean age was 37.4 years, the most frequent age group being 20–30 years (1200 respondents). Most participants were located in the United States (68.1%), 5.9% in Canada, 4.4% in the United Kingdom, 2.4% in Australia, 1.6% in Italy, with the rest distributed among a large number of countries worldwide with 1–40 respondents/country. Respondents were recruited to participate in a study on regret when visiting a procrastination-themed website.

### Material and Procedure

All respondents answered a questionnaire consisting of standard demographic questions followed by items from the complete DPS, GPS, AIP, and IPS scales. The DPS (Mann, 1982, unpublished; Mann et al., 1997) contains five items that primarily focuses on delay in planning and decision making, e.g., “I waste a lot of time on trivial matters before getting to the final decisions,” though has one item related to implementation, “Even after I make a decision I delay acting upon it” (DPS 2). Internal reliability for the DPS is relatively high, α = 0.70–0.83 (Mariani and Ferrari, 2012). The GPS (Lay, 1986) encompasses 20 items focusing primarily on implemental delay, e.g., “Even jobs that require little else except sitting down and doing them, I find that they seldom get done for days” (GPS 7). Two versions of the GPS exist, a general version and a version adapted for students specifically. The general version was used here. It has a good internal consistency, α = 0.86 (Lay, 1986). The AIP (McCown et al., 1989) contains a mix of items addressing decisional and implemental delay, as well as lateness (see **Table 2**). Test-retest reliability of this scale is relatively high, *r* = 0.71, as is internal consistency, α = 0.86 (Ferrari et al., 2005). The IPS (Steel, 2010) is a nine-item scale focusing on implemental delay, e.g., “I delay tasks beyond what is reasonable” (IPS 7). The IPS demonstrates good internal reliability, α = 0.91 (Steel, 2010). Of note, the PPS was not included as a separate scale, as this scale is composed of 12 items from the DPS, GPS, and AIP. Steel (2010) reported internal consistency of the PPS at α = 0.92. For discriminant validity purposes, respondents answered the five-item Satisfaction with Life Scale (SWLS; Diener et al., 1985). All items were answered on a common 1–5 scale, 1 = “Very seldom or not true of me,” 5 = “Very often true, or true with me.” All answered a total of 159 items. First, respondents answered the demographic questions, then the procrastination scales of the present study and finally the SWLS. Items were presented in fixed order, one scale at a time.

### Ethics Statement

Participation was voluntary, anonymous, and confidential. Participants read a consent form describing the nature and purpose of the study and then provided written informed consent before responding. No payment was provided. The project of which this study was a part received ethics approval from the Conjoint Faculties Research Ethics Board (CFREB) at the University of Calgary.

### Statistical Analyses

The item scores were first examined for skewness and kurtosis. Then multivariate normality was assessed for all scales, in particular multivariate kurtosis, which is important to parameter estimation in CFA (Byrne, 2008). Non-normality was apparent in each scale according to the Mardia skewness and kurtosis tests. Hence, we report the Satorra–Bentler scaled chi-square statistic which is robust to multivariate non-normality (Satorra and Bentler, 2001). Configural fits to the suggested models were evaluated in CFA according to the root mean square error of approximation (RMSEA), the Bentler comparative fit index (CFI), and the standardized root mean square residual (SRMR) (Byrne, 2001). Acceptable goodness of fit adopted the standard criteria of RMSEA < 0.08, CFI values in the 0.90–1.00 range, and SRMR < 0.08 (Brown, 2015; Kline, 2016). After having established acceptable configural baseline models for the PPS and IPS, those models were tested for measurement invariance over gender and age groups, using standard procedures to test for configural, metric, and scalar invariance (Byrne, 2001; Gregorich, 2006; Brown, 2015). Scales reflecting a single latent construct were also analyzed by IRT using the graded response model (GRM), focusing on parameter a (discrimination) and the difficulty parameter (e.g., Fraley et al., 2000). CFAs and IRTs were performed using the SEM and IRT modules in STATA 14.2^1^.

## Results and Discussion

### PPS Factor Structure

As discussed, four different factor models have been suggested for the PPS. These are shown in **Table 3** along with the CFA fit indices for the present data. As is seen in the table, the three-factor model for the PPS – items 1–3 measuring decisional procrastination, items 4–8 measuring implemental delay, items 9–12 measuring timeliness, and promptness – was superior to the other suggested models. As the one-factor model and the suggested three-factor models are nested, a Δ Chi squared comparison between these models indicates whether one model demonstrates a better fit (Brown, 2015). This difference was significant, Δ Chi squared = 2062.85, Δ *df* = 3, *p* < 0.001. Also, the Δ CFI between these models was 0.07, well above the 0.01 criterion suggested by Cheung and Rensvold (2002).

**Table 3:** For the PPS, confirmatory factor analysis (CFA) results for four suggested factor solutions.

Suggested models	Chi squared (S_B)	*df*s	RMSEA (S_B)	CFI (S_B)	SRMR
(1) One-factor (Steel)	3193.85 (2531.24)	54	0.118 (0.105)	0.888 (0.894)	0.049
(2) Two-factor (Rebetez)	1912.30 (1503.31)	43	0.102 (0.090)	0.926 (0.931)	0.042
(3) Two-factor (Rozental)	2328.45 (1841.95)	53	0.101 (0.090)	0.919 (0.923)	0.042
(4) Three-factor model	1131.00 (895.69)	51	0.071 (0.063)	0.961 (0.964)	0.032

*Satorra–Bentler corrected estimates in parentheses. (1) One-factor model (Steel, 2010); (2) two-factor model (PPS items 1–8 “voluntary delay,” and items 9–11 “observed delay”), ignoring item 12 (Rebetez et al., 2014); (3) two-factor model (Items 4–8 starting late, lagging behind, and wasting time on other things, and items 1–3 and 9–12 focusing on delayed decision making, not meeting deadlines, and missing appointments) (Rozental et al., 2014); (4) three-factor model: PPS items 1–3, 4–8, 9–12 (Svartdal et al., 2016)*.

**FIGURE 1:**
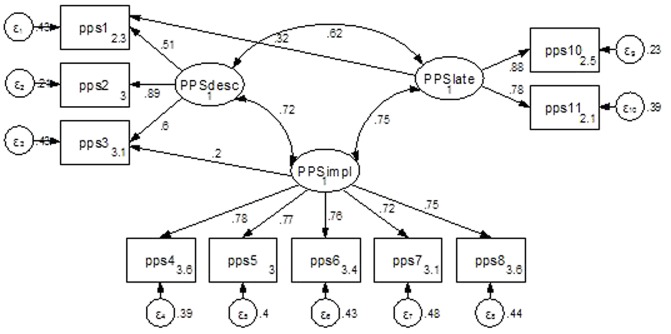
Final three-factor model for the Pure Procrastination Scale (PPS). PPS_desc_ = items 1–3, PPS_impl_ = items 4–8, PPS_late_ = items 9–12. Standardized estimates shown.

An examination of modification indices of the three-factor solution indicated a path to be added between the PPS factor timeliness/delay and PPS item 1. This connection is reasonable because this item explicitly addresses timeliness/lateness (“I delay making decisions until it’s too late”). Adding this path improved fit, RMSEA = 0.065, CFI = 0.968, SRMS = 0.029.^2^ In the next iteration of modification indices analysis, a path from PPS factor implemental delay and item 9 was suggested. This item (“I find myself running out of time”) has previously been argued to be conflated with the busyness construct and not procrastination *per se* (Steel, 2010; Svartdal et al., 2016) and recommended for deletion from procrastination scales. Deleting it improved overall fit, RMSEA = 0.062, CFI = 0.974, SRMS = 0.028. In the final iteration, modification indices indicated a path from PPS factor implemental delay and PPS item 3 (“I waste a lot of time on trivial matters before getting to the final decisions”), improving fit even more, RMSEA = 0.043, CFI = 0.989, SRMS = 0.015. In this model, shown in **Figure 1**, correlations between PPS factors were all < 0.75, indicating discriminant validity. As a more formal test of discriminant validity, we compared the squared correlations (SC) between factors with the average variance extracted (AVE) by the latent variables (Brown, 2015). All AVE values were higher than the SC values, indicating discriminant validity, and all AVE values were higher than 0.05, indicating convergent validity.

Addressing the PPS parts specifically, PPS items 4–8 should measure implemental delay satisfactorily. A CFA of these items indicated good fit, RMSEA = 0.064 (0.053), CFI = 0.991 (0.992), SRMR = 0.016. Regarding the IRT analysis of this construct, parameter a coefficients were > 2 for all items, item 7 demonstrating the lowest coefficient (2.15) and item 4 the highest (2.71). All items covered the range of the latent construct quite well, from -3 to +2, indicating that this short scale measures implemental procrastination well in individuals in the normal range of the latent trait. Note that the scale discriminates rather poorly in the higher end of the latent construct, speaking for cautious use in clinical settings. Test Information Function (TIF) and Item Information Functions (IFF) graphs are shown in Appendix. A corresponding examination of PPS items 9–12 (lateness/timeliness items from the AIP) indicated an excellent fit, RMSEA = 0.056 (0.049), CFI = 0.996 (0.997), SRMR = 0.011. The IRT demonstrated discrimination coefficients between 1.72 (item 12) and 3.50 (item 10). Examination of the TIF graph (Appendix) again indicated rather poor discrimination in the higher end of the latent construct. Finally, examination of the decisional part of PPS, items 1–3, demonstrated an excellent fit, RMSEA = 0.000, CFI = 1.00, SRMR = 0.000. The IRT discrimination coefficients ranged from 2.18 (PPS item 1) to 3.85 (item 2). Again, the TIF graph (Appendix) indicated less reliability in the higher end of the latent construct. In summary, the three-factor model of the PPS, as well as reduced models focusing on three unidimensional constructs, decisional, implemental delay, and lateness/timeliness, all appear to work well psychometrically.

Given the basic configural model of the PPS, we tested invariance across gender and age groups. Both gender and age differences (i.e., 30 years and above versus below) have been discussed repeatedly in the literature (e.g., Steel and Ferrari, 2013; Beutel et al., 2016), but as scalar measurement invariance is a prerequisite for meaningful comparisons of means over populations (Gregorich, 2006; Brown, 2015), conclusions about such differences cannot be settled until invariance has been established. As shown in **Table 4**, a multigroup men vs. women CFA indicated configural as well as metric invariance, but not scalar invariance. Hence, comparisons of PPS means with gender is problematic. Also note that configural fit was improved for participants > 30 years of age. The results further indicated that gender differences appeared in the decisional and lateness parts of the PPS (*z* = -3.65 and -6.58, *p* < 0.000), but not in the implemental part (*z* = -0.28, *p* = 0.777). Hence, invariance tests of PPS items 4–8 demonstrated a similar pattern to that of the complete PPS, with the important exception that scalar equivalence was now observed for the age group > 30 (see **Table 5**). In summary, the PPS results indicate that the complete scale does not attain full invariance across gender, and furthermore the model fit was better for participants greater than 30 years of age. For the reduced PPS (items 4–8, i.e., the implemental part), gender differences were minimal and these items also demonstrated full measurement invariance for participants over 30 years, indicating that this part of the scale permits comparisons of means scores for adults.

**Table 4:** Pure Procrastination Scale invariance tests, gender and age.

	*N*	χ^2^ (*df*)	RMSEA	Diff χ^2^ (*df*), *p*	CFI
Gender					
Men	1750	139.606	0.046		0.988
Women	2357	164.809	0.044		0.989
Multigroup analysis					
Configural		304.41 (60)	0.04		0.99
Metric		313.57 (69)	0.04	9.16 (9), 0.42	0.99
Scalar		460.36 (79)	0.05	146.78 (10), 0.00	0.98
Age groups					
Age < 30	1594	172.058 (30)	0.055		0.981
Age > 30	2574	124.308 (30)	0.035		0.994

**Table 5:** Pure Procrastination Scale (Items 4–8) invariance tests, gender and age.

	*N*	χ^2^ (*df*)	RMSEA	Diff χ^2^ (*df*), *p*	CFI
Gender					
Men	1750	31.159 (5)	0.055		0.993
Women	2357	68.144 (5)	0.073		0.988
Multigroup analysis					
Configural		99.30 (10)	0.07		0.99
Metric		100.64 (14)	0.05	1.34 (4), 0.86	0.99
Scalar		113.67 (19)	0.05	13.03 (5), 0.02	0.99
Age groups					
Age < 30	1594	54.003 (5)	0.078		0.983
Age > 30	2574	42.590 (5)	0.054		0.994
Gender, age > 30					
Configural		54.79 (10)	0.06		0.99
Metric		55.23 (14)	0.05	0.45 (4), 0.98	0.99
Scalar		58.92 (19)	0.04	3.69 (5), 0.60	0.99

### IPS Factor Structure

The IPS is hypothesized to measure a single construct, “irrational delay,” and the present data indicates that it does, RMSEA = 0.075 (0.066), CFI = 0.971 (0.973), SRMR = 0.032. In accordance with prior findings (Svartdal et al., 2016), modification indices indicated that the reversed items should be correlated. This resulted in an improved fit, RMSEA = 0.058, CFI = 0.984, SRMR = 0.019. Omitting the reversed items improved fit slightly. These analyses thus support the hypothesis that the IPS confirms to a single latent construct, implemental or irrational delay. Omitting the reversed items improves fit indices and provides an instrument that is more easily administered and scored. Supporting this, the IRT indicated good parameter a (discrimination) coefficients for all procrastination-consistent items (range 2.24–3.14), item 4 being lowest but covering the higher range of the latent construct better. The reversed items – and particularly items 2 and 9 – demonstrated the lowest coefficients (item 9 = 1.41; item 2 = 1.49). As for the PPS subscales, the TIF graph (Appendix) indicated rather poor discrimination in the higher end of the latent construct.

Testing IPS measurement invariance over gender and age groups indicated somewhat better fit for age over 30 years, but as is apparent from **Table 6**, even in the older group scalar invariance did not appear, indicating that care should be taken in comparing mean IPS scores between genders and age groups.

**Table 6:** Irrational Procrastination Scale invariance tests, gender and age (IPS reversed items not included).

	*N*	χ^2^ (*df*)	RMSEA	Diff χ^2^ (*df*), *p*	CFI
Gender					
Men	1750	66.259 (9)	0.060		0.991
Women	2357	107.792 (9)	0.068		0.989
Multigroup analysis					
Configural		174.05 (18)	0.06		0.99
Metric		197.56 (23)	0.06	23.51 (5), 0.00	0.99
Scalar		289.82 (29)	0.05	92.26 (6), 0.00	0.99
Age groups					
Age < 30	1594	103.455 (9)	0.081		0.983
Age > 30	2574	77.951 (9)	0.055		0.994
Gender, age > 30					
Configural		85.44 (18)	0.05		0.99
Metric		94.31 (23)	0.05	8.87 (5), 0.11	0.99
Scalar		128.90 (29)	0.05	34.59 (6), 0.00	0.99

### Relation between PPS and IPS

Given that items 4–8 of the PPS measure implemental or “irrational delay,” this part of PPS should correlate highly with IPS, whereas the two other factors of the PPS should demonstrate more moderate correlations. As is seen from **Table 7**, this was the case, *r* = 0.83 vs. 071 and 0.76. Further, IPS and PPS item means 4–8 should be comparable, and for the present sample they were, at 3.62 in both cases. These results indicate that PPS items 4–8 and IPS address the same unidimensional construct, implemental delay.

**Table 7:** Means, standard deviations, internal consistencies (Cronbach’s alpha) and correlations between the procrastination scales as well as the Satisfaction with Life Scale (SWLS).

Measure	*M*	*SD*	α	1	2	3	4	5	6	7	8
(1) DP	3.06	0.98	0.90	1.00							
(2) AIP	2.97	0.80	0.89	0.61	1.00						
(3) GPS	3.25	0.69	0.90	0.70	0.82	1.00					
(4) IPS	3.62	0.83	0.91	0.69	0.72	0.79	1.00				
(5) PPS	3.34	0.86	0.92	0.82	0.82	0.87	0.87	1.00			
(6) PPS_1-3_	3.15	0.97	0.83	0.97	0.62	0.70	0.71	0.84	1.00		
(7) PPS_4-8_	3.62	0.89	0.87	0.65	0.68	0.84	0.83	0.92	0.68	1.00	
(8) PPS_9-12_	3.13	1.04	0.85	0.66	0.86	0.75	0.76	0.90	0.66	0.72	1.00
(9) SWLS	3.04	0.98	0.90	-0.40	-0.36	-0.37	-0.41	-0.40	-0.41	-0.30	-0.39

### The GPS Factor Structure

As mentioned, Lay (1986) proposed the GPS as a scale measuring a unidimensional construct procrastination, but Argiropoulou and Ferrari (2015) suggested a two-factor solution (*delay* and *procrastination domains*), and a German study, testing the student version of the GPS, proposed a reduced version – GPS-K – consisting of items 1, 2, 7, 12, 14, 15, 18, 19, and 20 (Klingsieck and Fries, 2012). These items (except items 2 and 14) are identical in the general version of GPS. In the present study, the one-factor model for the complete GPS did not demonstrate a good fit, RMSEA = 0.088, CFI = 0.824, SRMS = 0.058; the two-factor model indicated somewhat better fit, RMSEA = 0.070 (0.064), CFI = 0.89 (0.89), SRMS = 0.058. In both cases, however, the CFI criterion was not acceptable. The German reduced model, excluding items 2 and 14, indicated a somewhat better fit, although not acceptable, RMSEA = 0.088 (0.076), CFI = 0.96 (0.97), SRMS = 0.031.

### AIP Factor Structure

As the other general procrastination scales discussed, the AIP is hypothesized to measure a single latent construct, procrastination, and Mariani and Ferrari (2012) reported support for a single-factor latent model in an Italian sample. The present data did not indicate a good fit for this model, RMSEA = 0.135, CFI = 0.761, SRMS = 0.076. Mariani and Ferrari (2012) reported an even better fit when errors of items 13, 4, 7, and 8 were allowed to correlate. This is theoretically reasonable, as these items concern things to do before a deadline. Again, this model did not improve fit indices in the present data. Thus, the present data did not support either of the suggested factor solutions for the AIP. Analysis of individual items indicates that the AIP focuses on rather different aspects of procrastination (see **Table 2**), which in part may explain why this scale did not do well in the CFA analyses.

### DPS Factor Structure

The DPS demonstrated a poor fit for a one-factor solution, RMSEA = 0.259, CFI = 0.916, SRMS = 0.076. Modification indices suggested correlations between errors for items 4 and 5 (two items with quite similar wording), and then between items 1 and 2. This model indicated an excellent fit, RMSEA = 0.017, CFI = 1.000, SRMS = 0.002.

### Relation between the Scales

**Table 7** shows the Cronbach’s alphas, correlations and mean scores for the DPS, AIP, and GPS scales as well as for the IPS and PPS (complete and subscales). SWLS is also included to evaluate divergent validity. Overall, good convergent validity was observed between the procrastination scales, and divergent validity to the SWLS was apparent for all instruments. Note that the complete PPS correlates highly with the GPS, AIP, and DPS (all correlations > 0.81), making the PPS a briefer alternative to these scales. Also note that the DPS total scale correlates very highly with the first factor of the PPS, *r* = 0.97, effectively making the DPS part of the PPS equivalent to the complete DP scale. Similarly, the complete AIP correlates highly with the PPS factor containing AIP items, *r* = 0.86, making these four items comparable to the complete AIP.

**FIGURE 2:**
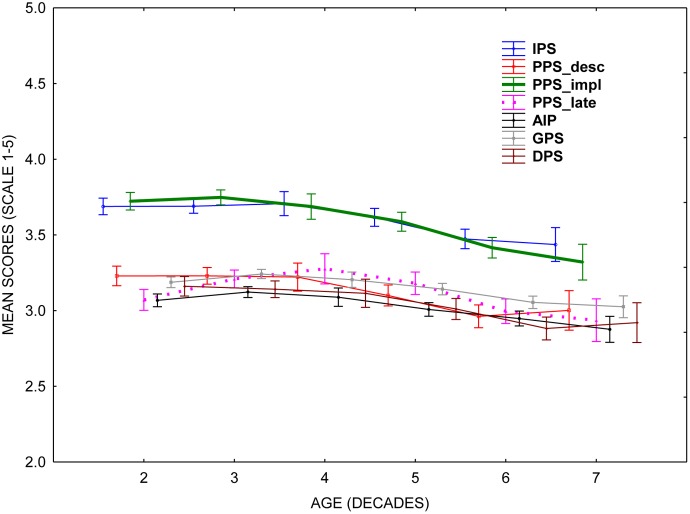
Relations between age (decades) and the procrastination scales. See text for explanation.

As scalar measurement invariance was demonstrated in the PPS 4–8 subscale for age > 30, we plotted mean PPS 4–8 subscale scores over age (decades). This is shown in **Figure 2**. The figure indicates a slight reduction of procrastination over age decades 40–70, supporting the view that procrastination decreases with age (Steel and Ferrari, 2013; Beutel et al., 2016). For illustrative purposes we also plotted the other scales and subscales in the figure. Note that all scales agree to an overall decrease over decades, one deviation being the PPS items 9–12 subscale, indicating that timeliness/lateness forms of procrastination increase until 40 years of age, then decreases. However, this result must be interpreted with great caution, as scalar invariance was not observed for other scales or subscales.

## General Discussion

The present study examined the psychometric properties of five prevalent procrastination scales, with a main focus on the PPS and IPS. All scales were assessed with CFA and – for scales/subscales measuring one-dimensional constructs – also with IRT. For the PPS, the results indicated that this scale conforms to a three-factor solution corresponding to the three different scales the PPS is based on, measuring *decisional procrastination*, *delay in implementation*, and *timeliness/lateness*. The three PPS subscales enable this scale to measure three facets of procrastination in much the same way with 12 items as is achieved by three separate scales with 39 items. This is a substantial practical advantage as well as a psychometrically sounder solution, as the reduced set of items selected for the PPS were shown to demonstrate better CFA fit indices compared to the full set of items of the individual DPS, GPS, and AIP scales.

Simply collapsing the 12 PPS items into one score implies a potential loss of information. Thus, the first part of the PPS, measuring decisional procrastination, correlated very highly with the full DP scale, and the last part of PPS, measuring timeliness/lateness, correlated very highly with the complete AIP scale. Importantly, the implemental part of the PPS (items 4–8; PPS_impl_) appears to measure irrational delay in much the same way as does the IPS, and thus represents an even “purer” version of the PPS in measuring irrational delay. Additionally, this part of PPS also correlates very highly with the complete GPS, suggesting that this 20-item scale might be reduced to a 5-item scale without loss of information. Collapsing the three facets of the PPS into one score also masks the substantial mean differences in scores between the implemental part of PPS (items 4–8) and the two other facets, the former being consistently higher compared to the two others (see **Figure 2**). Finally, the lateness part of PPS (items 9–12) may be more sensitive to cultural differences compared to the two other facets (Svartdal et al., 2016) and also appears to relate to age differences differently from all the other scales/subscales examined in this study.

The IPS conforms to a one-factor solution, the construct measured being very similar to the implemental part of the PPS. The IPS includes three reversed items. In agreement with prior findings (Rozental et al., 2014; Svartdal et al., 2016), the analyses indicate that these items can be deleted from the scale without significant loss of information.

The two scales demonstrating acceptable fits to suggested factor structures, PPS and IPS, were examined for measurement invariance across gender and age (i.e., above and below 30 years of age). Neither of these scales demonstrated full scalar invariance. As scalar invariance is required for meaningful comparisons between population means, gender and age differences cannot be assessed unless a given instrument is demonstrated to satisfy measurement invariance requirements. However, note that the implemental part of the PPS (items 4–8) seems to perform better compared to items related to decisional procrastination (items 1–3) and timeliness/lateness (items 9–12), and full scalar invariance for the PPS_impl_ was observed for participants greater than 30 years.

The present results are based on answers from many nations, albeit with English as a common language. Hence, we cannot unambiguously assess cultural or national differences. We believe, however, that the present results, especially regarding the PPS and IPS, are quite robust. Thus, the conclusions from the present paper regarding PPS and IPS factor structures are very similar to prior findings in a comparison of these scales in Finland, Germany, Italy, Norway, Poland, and Sweden (Svartdal et al., 2016), and the suggested PPS_impl_ subscale (items 4–8) conforms well to recent findings in a German representative community study (Klein et al., 2017). That study proposed a shortened version of the GPS-K (Klingsieck and Fries, 2012) consisting of five items. These items are identical to the PPS_impl_ items proposed in this paper except that the German version, being based on the student version of the GPS, uses the item “I do not do assignments until just before they are to be handed in” (GPS – student, item 2) rather than “In preparing for some deadline, I often waste time by doing other things” (GPS item 12; PPS item 4; see **Table 1**). In the present study, the latter item demonstrated excellent item properties (Appendix) and appears to be more appropriate as an item measuring procrastination in the general population. However, future studies should examine these scales, both in item-level analyses and in cross-cultural comparisons. At present, the implemental part of the PPS and the IPS seem to be the best available candidates for assessing procrastination over different languages and cultures.

The scales examined in this study all differentiate procrastination well for low and medium ranges of the construct, but appear to measure less reliably in the higher end of the construct. This implies that measurement of high levels of procrastination, for example in clinical cases, is error prone. Hence, assessment of procrastination in clinical settings should be supplemented by other measures (e.g., depression and anxiety) to ensure sufficient validity and reliability. Another issue of importance for future research is to establish more objective and reliable measures that can supplement or even replace self-report measures (Gröpel and Steel, 2008). This is complicated by procrastination having an inherent subjective component as delays are only irrational if they are inconsistent with a person’s internal preferences. What may be a procrastination for one might easily not be for another. Still, the delay in implemental procrastination can be observed and at times this may be less ambiguously connected to procrastination (e.g., seeking treatment for a dire medical condition). This should help identify those who delay somewhat trivially, but judge themselves harshly. Such people would be best described as perfectionists rather than procrastinators, which has a different etiology and treatment recommendations (Steel and Klingsieck, 2016).

## Author Contributions

PS was responsible for data collection. FS wrote the draft and did the statistical analyses. Both authors did final editing of the manuscript.

## Conflict of Interest Statement

The authors declare that the research was conducted in the absence of any commercial or financial relationships that could be construed as a potential conflict of interest.

## Footnotes

1 www.stata.com

2 The Satorra–Bentler scales estimates are not available when performing modification analyses in Stata 14.2.
