# Supplementary material for: Irrational Delay Revisited: Examining Five Procrastination Scales in a Global Sample
Source: Front Psychol. 2017 Nov 3;8:1927. doi: 10.3389/fpsyg.2017.01927 (PMC5676095; doi:10.3389/fpsyg.2017.01927)
Supplement: Supplementary file 1 [file Appendix.docx]

Appendix

IRT Test Information Function (TIF; standard errors shown) and Item Information Functions (IFF) for the three PPS factors and IPS


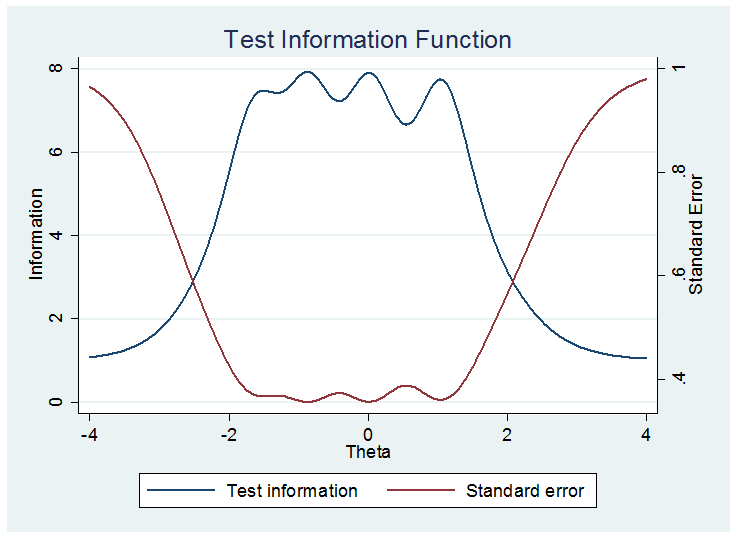


PPS 1-3 (decisional)


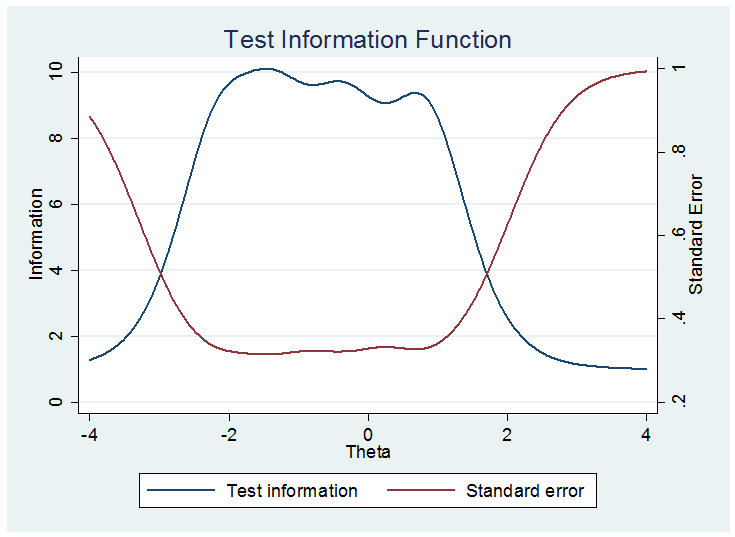

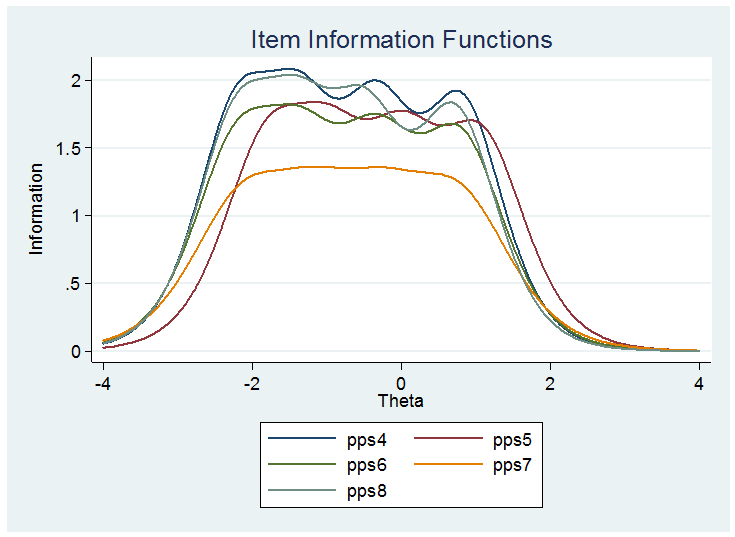


PPS items 4-8 (implemental)


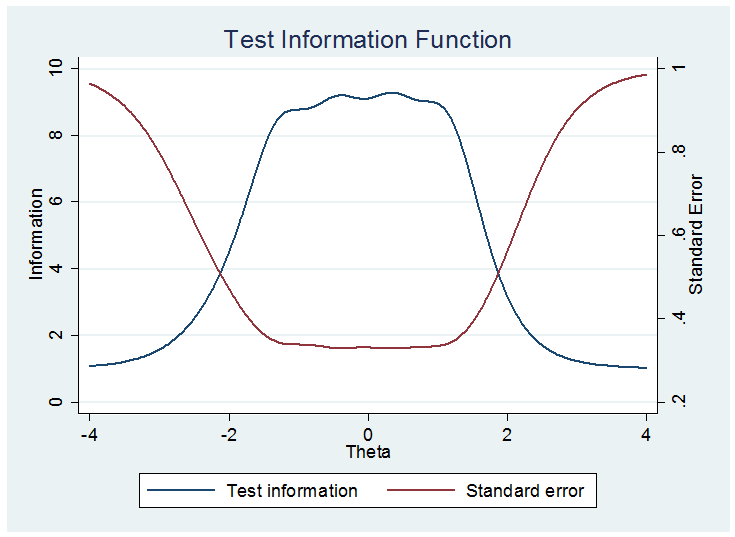

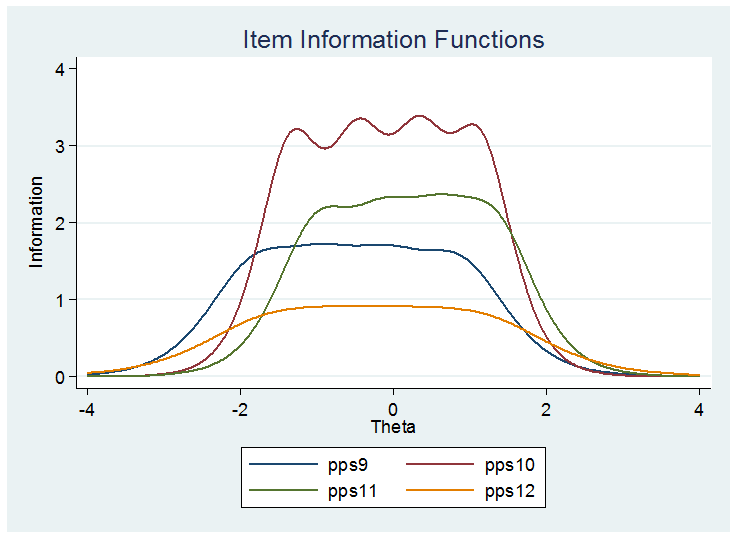


PPS items 9-12 (lateness)


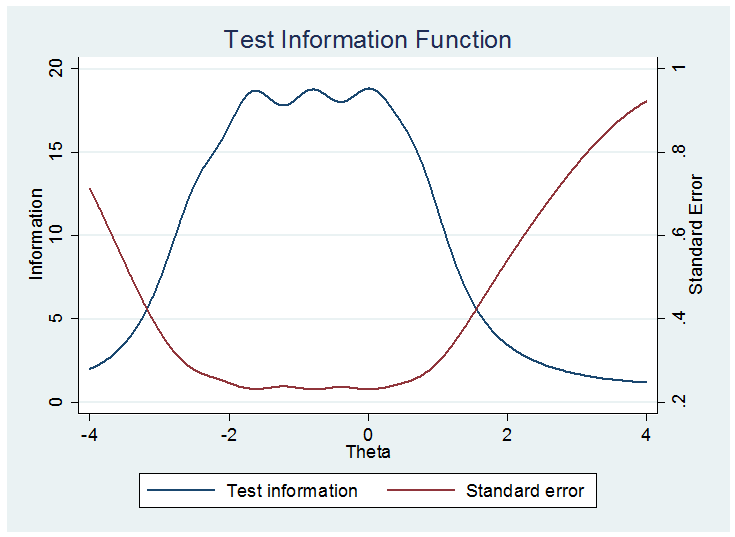

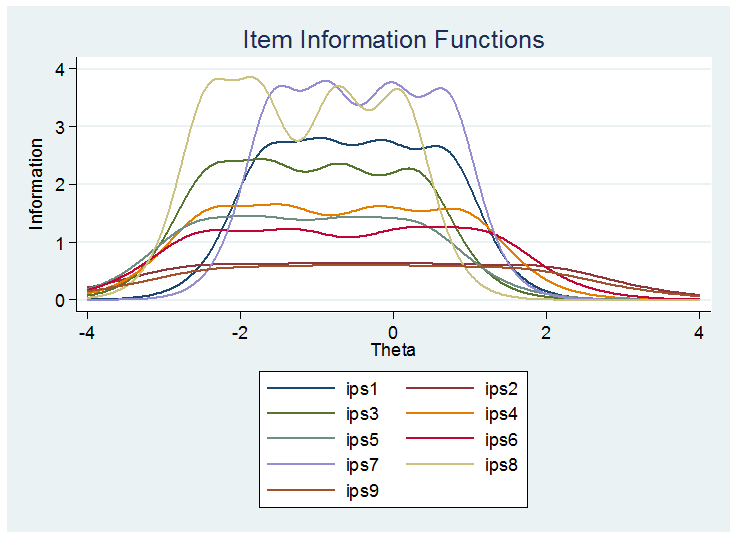


IPS
